# Value assessment of medicinal products by the Italian Medicines Agency (AIFA) and French National Authority for Health (HAS): Similarities and discrepancies

**DOI:** 10.3389/fmedt.2022.917151

**Published:** 2022-09-05

**Authors:** Entela Xoxi, Rossella Di Bidino, Serena Leone, Andrea Aiello, Mariangela Prada

**Affiliations:** ^1^ Intexo SB Rome Italy; ^2^ Postgraduate School of Health Economics and Management (ALTEMS), Università Cattolica del Sacro Cuore, Rome, Italy; ^3^ Health Technology Assessment Unit, Fondazione Policlinico Universitario Agostino Gemelli Istituto di Ricovero e Cura a Carattere Scientifico, Rome, Italy; ^4^ Patient Access Unit, Intexo SB Milan Italy

**Keywords:** therapeutic added value, innovation, improvement of effective clinical benefit, reimbursement, agreement, framework, health technology assessment (HTA), payer

## Abstract

The evaluation of pharmaceutical innovation and therapeutic value is an increasingly complex exercise for which different approaches are adopted at the national level, despite the need for standardisation of processes and harmonisation of public health decisions. The objective of our analysis was to compare the approaches of the AIFA (*Agenzia Italiana del Farmaco*) and the HAS (*Haute Autorité de Santé*) in assessing the same medicinal products. In Italy, the 1525/2017 AIFA Deliberation introduces a transparent scheme for the evaluation of innovative status (innovative, conditional, not innovative) based on the therapeutic added value (TAV), therapeutic need, and quality of evidence. In contrast, in France, the HAS makes judgements using the effective clinical benefit (*Service Médical Rendu*) and improvement of effective clinical benefit (*Amélioration du Service Médical Rendu*, ASMR). This analysis focused on medicinal products evaluated both by the AIFA and by the HAS from July 2017 to September 2021. Similarities between AIFA and HAS evaluations were investigated in terms of the TAV, recognition of innovativeness, and the ASMR. Both total and partial agreements were considered relevant. Therefore, raw agreement, Cohen's kappa (weighted and unweighted), and Bangdiwala's B-statistic were estimated. A total of 102 medicinal products were included in this study. Out of these, 38 (37.2%) were orphan drugs, while 56 (54.9%) had a clinical indication for the treatment of cancer. The AIFA and HAS reached a higher level of agreement on the innovativeness status compared with the TAV. A moderate total agreement emerged in the recognition of innovativeness (*k* = 0.463, *p*-value ≤0.0001), and partial agreement was substantial (equal weight *k* = 0.547, squared *k* = 0.638), while a lack of agreement resulted in a comparison of the TAV according to the AIFA and the ASMR recognised by the HAS. Indeed, whereas the AIFA determined the TAV to be important, the HAS considered it to be moderate. In addition, whereas the AIFA identified a bias towards a moderate TAV, the HAS identified a bias towards a minor ASMR. A higher level of agreement was reached, both on the TAV and on innovative status, for less critical medical products (non-cancer-related, or non-orphan, or with a standard European Medicines Agency approval). These results underline the importance of implementing European procedures that are more broadly aligned in terms of value definition criteria.

## Introduction

The European evaluation of medicinal products is followed by an evaluation of individual countries by health technology assessment (HTA) agencies or payers, who primarily evaluate the relative effectiveness analysis and decide how to introduce the product on the national market. As is already established, the variability of the decisions of the individual Member States and the United Kingdom has been the subject of comparison for several years. There have been numerous projects that have tried to harmonise the assessment of new technologies. The latest proposal of the European Commission on clinical joint assessment (CJA) launched in 2018 has finally been granted the approval of the European Parliament with the new EU Regulation of the HTA with the decision to start a voluntary CJA path on medicines involving the following: (a) rare diseases (medicines with orphan designation); (b) oncological area; and (c) advanced therapy medicinal product (1).

The assessment of health technologies is, therefore, a key tool used in the decision-making process in health policies. Although various initiatives have been launched in recent years (2–6), ratings still differ significantly between European countries (7, 8) in terms of the variability of the approach of the legislative framework that regulates the activities of the national agencies. It is also important to consider the ability of individual states to adapt their government systems to the technological revolution currently taking place in the world of health and pharmaceuticals.

Variability across countries in assessment of the therapeutic value of new medicines to support reimbursement decisions is a well-known problem, but it is not so easily addressed. Understanding and addressing that variability at a more granular level is thus important from the perspective of equal patient access to (innovative) treatments across Europe. This article will investigate the value assessments according to two European authorities: those of the Italian Medicines Agency (Agenzia Italiana del Farmaco, AIFA) and the French Agency (Haute Autorité de Santé, HAS). The decision to choose Italy and France is justified as there are several similarities in the way that they evaluate drugs. Both agencies consider in their evaluation the therapeutic benefits of the medicine and also the actual improvement that the medicinal product brings compared with the current standard of care (SoC). However, the ways in which they investigate these criteria differ; hence, we have decided to analyse them and assess the similarities and discrepancies between the approaches adopted by the two agencies.

### Background

The authority responsible for the evaluation of medicinal products in Italy is the Italian Medicines Agency (AIFA). The AIFA governs pharmaceutical expenditure and monitors the life cycle of medicine to ensure its efficacy, safety, appropriateness, and availability across the national territory (9). The evaluation and approval of a new drug are managed by the Technical Scientific Commission (*Comitato Tecnico-Scientifico*, CTS) and the Price and Reimbursement Committee (*Comitato Prezzo e Rimborso*, CPR). The CTS evaluates applications for national (and community) registrations, offers an advisory opinion, and classifies medicines for reimbursement purposes. The CPR, consequently, carries out negotiations with pharmaceutical companies in order to set the price of drugs reimbursed by the National Health System (*Sistema Sanitario Nazionale*, SSN) and, in collaboration with the CTS, determines their reimbursement (9). The 2017 Budget law (No. 232/2016) (10) establishes two funds of 500 million euros each for reimbursement to the region for the purchase of innovative medicines and innovative oncological medicines. Article 1 paragraph 402 requires the AIFA to establish the criteria for the classification of innovative drugs (oncologic and not) (11). The innovation evaluation model [at label (European Medicines Agency, EMA) therapeutic indication] proposed by the AIFA consists of a multidimensional approach based on three criteria (12): (1) unmet therapeutic need, (2) therapeutic added value (TAV), and (3) quality of evidence (GRADE methodology) (13–15). The therapeutic need is conditioned by the therapies available for the specific disease in question, and it indicates to what extent the new therapy is necessary to satisfy the therapeutic needs of the patients (12). It is articulated according to five levels ranging from Maximum (*absence of therapeutic options for the indication in question*) to Absent (*existence of therapeutic options for the indication in question, capable of modifying the natural history of the disease, and having a favourable safety profile*). The TAV represents the extent of the clinical benefit provided by the medicinal product on outcomes recognised and validated as clinically relevant (Table 1) (12). Finally, the quality of the evidence concerns the robustness of the clinical studies, while the evaluation uses the GRADE method (12) and is divided into four levels (16): (1) High, (2) Moderate, (3) Low, and (4) Very low.

**Table 1:** AIFA therapeutic added value and HAS ASMR scores.

AIFA		HAS	
*Score*	TAV	*Score*	ASMR
*Maximum*	The medicinal product is able to heal or modify the course of the disease. Moreover, it has greater efficacy in terms of its clinically relevant outcomes than the therapeutic alternatives available	*(I) Improvement of the maximum benefit*	Important therapeutic advance and is assigned only for medicinal products that lead to a decrease in mortality in severe disease
*Important*	The medicinal product is capable of modifying the natural history of disease in a subpopulation of patients, or provides a clinically relevant advantage (e.g. quality of life and disease-free interval)	*(II) Major benefit improvement*	Significant improvements in terms of therapeutic efficacy and/or reduction of side effects
*Moderate*	The medicinal product leads to moderate improvement or to greater efficacy in some subpopulations of patients or on surrogate outcomes, and with limited effects on quality of life	*(III) Moderate benefit improvement*	Moderate improvements in terms of therapeutic efficacy and/or reduction of side effects
*Poor*	The medicinal product has demonstrated greater efficacy on non-clinically relevant outcomes or has low efficacy. Furthermore, it has minor advantages over the therapeutic alternatives available	*(IV) Minor benefit improvement*	Minor progress in terms of therapeutic efficacy and/or reduction of side effects.
*Absent*	The medicinal product lacks an additional clinical benefit over the therapeutic alternatives available	*(V) No improvement in clinical benefit*	No improvement in clinical benefit.

AIFA, Italian Medicines Agency; HAS, Haute Autorité de Santé; TAV, therapeutic added value; ASMR, Amélioration Du Service Médical Rendu.

The possible label therapeutic-indication-level judgments of the AIFA innovativeness recognition are reported in Table 2 (12).

**Table 2:** Description of the characteristics associated with the AIFA innovative judgment assigned to the medicinal product.

Innovativeness recognition	Duration of the judgment of innovativeness	Allocation of economic benefits	Access to the fund for innovative or cancer innovative drugs	Automatic inclusion in regional therapeutic formularies
Full	36 Months	Yes	Yes	Yes
Conditional	18 Months	No	No	Yes
Failed	NA	No	No	No

Market access in France is regulated by the Ministry of Health (*Ministre Santé et Sécurité Sociale*), which sets a reimbursement level and negotiates a price that reflects the added therapeutic value of a product. The committee responsible for determining the prices of medicinal products is the Committee for the Pricing of Medicinal Products (*Comité Economique des Produits de Santé*, CEPS), which is made up of officials from different ministries. The 2004 reform led to the creation of two new institutional bodies: the French National Health Authority (Haute Autorité de Santé, HAS) and the National Union of Health Insurance (*Union Nationale des Caisses d’Assurance Maladie*, UNCAM). The UNCAM is the body responsible for uniting the main health insurance funds and is also responsible for determining which medicinal products to reimburse with their relative reimbursement rates (17, 18).

The HAS is an independent public body and its duties include providing adequate information to regulatory bodies in order to set prices and encourage good practices and ensuring the correct use of medicinal products (17). The Transparency Commission (*Commission de la Transparence*, CT) is composed of independent scientists united by the goal of evaluating medicinal products in terms of both the level of innovation that the drug brings to the market and the importance of the drug for the health of citizens. The CT uses two criteria to evaluate medicinal products: the *Service Médical Rendu* (SMR), representing the actual clinical benefit, and the *Amélioration du Service Médical Rendu* (ASMR), representing the improvement of the effective clinical benefit (17). Finally, the combination of the scores of the two criteria is used by the CT to determine the reimbursement level for the medicinal product. The reimbursement rate will be defined according to the actual benefit level (SMR): Important (65%), Moderate (30%), Mild (15%), and Insufficient (not included on the positive list) (17, 19, 20).

The SMR is used to determine the actual clinical benefit of the medicinal product in question. To express the SMR judgment, the following five criteria are considered: (1) the severity of the disease and its impact on morbidity and mortality, (2) the purpose of the drug, (3) the therapeutic alternatives, (4) its place in therapy, and (5) any public health considerations (such as disease burden, community health impact, quality of clinical trials, etc.) (17, 19, 20). The ASMR, unlike the SMR, is used to determine the degree of the actual clinical benefit. To express the ASMR judgment, the HAS considers the added therapeutic value that the medicinal product brings compared with the therapeutic alternatives for the same therapeutic indication and the improvement it brings. Therefore, the ASMR judgment answers the question of whether the drug improves the clinical benefit for patients compared with the current SoC (17, 19, 20). The ASMR judgment is represented by a score on a scale of 1 to 5 (Table 1) (17, 19, 20).

The ASMR and SMR ratings described above are determined simultaneously. Once determined, the manufacturer enters into negotiations with the *Comité Economique des Produits de Santé* (CEPS) to establish the reimbursement price and rate for innovative outpatient medicines. The two judgments are, therefore, used not only to evaluate the value/innovativeness of medicinal products but also to determine their price and reimbursement (17, 19, 20). The ASMR is used to determine the price of the medicine. Table 3 shows the price levels corresponding to the ASMR score assigned to the medicinal product. The SMR, by contrast, is used to assess whether the medicinal product should be reimbursed. The SMR judgment and its consequent reimbursement are valid for 5 years, after which the medicinal product will be re-evaluated on the basis of the new data provided to the *Commission de la Transparence* (17, 19, 20).

**Table 3:** Price level corresponding to the ASMR score assigned to the medicinal product under consideration.

ASMR score	Price level corresponding
V	The drug is reimbursed only if the costs are lower than the comparators
IV	The assessment takes into account the target population of the new drug. If the new drug targets the same population as the comparator, a fair price is desirable. The price may be higher than a comparator if the new drug has a better effect in a smaller population
I–III	Faster access (price notification instead of negotiation) and price consistency with the rest of Europe

## Objective

Despite methodological differences, investigating the similarities in the AIFA and HAS appraisals could provide a helpful insight into the consequences of the alignment or lack of it on the decisions of the two agencies. The aim of our analysis was, therefore, to investigate and describe the current status of the AIFA appraisals with regard to the TAV and the recognition of innovativeness (updated September 2021) and of the *Amélioration Du Service Médical Rendu* (ASMR) and the *Service Médical Rendu* (SMR) published by the HAS in order to grasp the differences and agreements in the judgments between the two agencies.

## Material and methods

A database was created by extracting the data from the AIFA innovation reports from the AIFA web page (21). The assessments included the period from July 2017 (start of AIFA report publications) to September 2021 (latest data available before processing). Each AIFA innovation report includes evaluation by a single criterion. Subsequently, the SMR and ASMR ratings were extracted from the HTA reports from the HAS web page (20). The extracted data include all the medicinal products on the AIFA list.

Microsoft Office Excel program was used to create the database.

### Data extraction

Overall, 127 HAS and AIFA reports on the same medicinal products with same label therapeutic indication were analysed. For each of them, the following were reported: AIFA's opinion of innovativeness’ recognition, orphan designation (22), EMA approval (23–25), anatomical therapeutic chemical classification (ATC) level, cancer/non-cancer drug, curative/non-curative/prophylaxis indication, reference population, type of therapy (monotherapy, combo, or add-on), AIFA innovation judgment, year in which the innovation was granted by the AIFA, AIFA TAV, AIFA quality of evidence judgment, National Health Service (NHS) reimbursement (both agencies), year of resolution in *Gazzetta Ufficiale*, presence of an AIFA registry, HAS ASMR judgment, HAS SMR judgment, and year of assignment of HAS ASMR judgment.

### Data comparison

The similarities between AIFA and HAS evaluations were investigated according to the following variables: AIFA therapeutic added value and innovativeness status; HAS effective clinical benefit. Table 4 reports adopted criteria to compare AIFA and HAS decisions. To compare the AIFA innovation scores with AIFA's ASMR ratings, the ratings were grouped as follows: (a) AIFA full innovativeness—ASMR I–III, (b) conditional innovation—ASMR IV, and (c) non-innovativeness—ASMR V and ASMR NA.

**Table 4:** Criteria to compare AIFA and HAS decisions.

	AIFA	HAS
Therapeutic added value	Maximum	ASMR I (Maximum)
	Important	ASMR II (Important/Significant)
	Moderate	ASMR III (Moderate)
	Poor	ASMR IV (Minor)
	Absent	ASMR V (Absent)
	NE	ASMR NA (Insufficient SMR)
Innovativeness judgment	Innovative	ASMR I–II–III
	Conditional	ASMR IV
	Not innovative	ASMR V + ASMR NA (Insufficient SMR)
Reimbursement status	Yes	SMR: Important (65%), Moderate (30%), Mild (15%)
	No	SMR: Insufficient (not included on the positive list)

AIFA, Italian Medicines Agency; ASMR, Amélioration du Service Médical Rendu; SMR, Service Médical Rendu; HAS, Haute Autorité de Santé.

The rationale is based primarily on the similar wording expressed by the two agencies on the therapeutic added value. Second, the level of innovation recognised by the AIFA incorporates other fundamental elements such as the therapeutic need. After all, ASMR of the HAS expresses whether the drug improves the clinical benefit for patients compared with the current SoC. Although obvious, both opinions are specific for therapeutic indication, so it confirms the similarity of the technical operations by the two agencies. Both agencies express their opinions: on superiority or inferiority clinical trial results, on the endpoints used, criticality on surrogate endpoints or lack of overall survival (OS) data (for cancer drugs), or long-term data plus tolerability. Both, and with a view to contextualising the therapeutic indication, summarise the clinical condition keeping in mind the therapeutic alternatives (SoC), identifying the place in therapy of the product.

AIFA's ASMR rating (a–c), although questionable, takes into consideration the data (Supplementary Table S2): there are 4 HAS ASMR II (all AIFA fully innovative), 18 ASMR III (of which 16 are fully innovative, 1 is conditional, and 1 is non-innovative recognised by the AIFA), 47 ASMR IV (of which 16 are fully innovative, 22 are conditional, and 9 are non-innovative recognised by the AIFA), and 24 ASMR V (of which 1 is fully innovative, 6 are conditional innovative, and 17 are non-innovative). There are no ASMR I. Based on these figures, our rating appears reasonable.

### Statistical analysis

Our final database included details on selected medicinal products, plus information on innovative status. Information on the reimbursement decisions was collected, although it was not subject to our analysis. First, descriptive statistics were conducted. Quantitative data were expressed as frequency and percentage. Contingency tables were then used to analyse the associations between the AIFA innovation ratings/scores and the TAV with those of HAS ASMR.

To investigate the association between the categorical variables, Fisher's exact test was used, while concordance was assessed as raw agreement (%), unweighted and weighted Cohen's kappa, and Bangdiwala's B-statistic.

With the raw agreement is reported the percentage of cases in which the two agencies made the same judgment (according to the criteria we adopted to compare the AIFA and HAS judgments). In other words, it is the percentage of cases along the diagonal of the 6 × 6 table for the TAV and 3 × 3 table for innovativeness. Unweighted kappa considered all disagreements equally, but weighted kappa assigned a different weight to disagreements according to the magnitude of the discrepancy. For categorical variables, as in our case, weighted kappa provided the most appropriate information (26). To interpret kappa statistics, the criteria reported in Altman (1991) (26) were adopted (Supplementary Table S1). The Bangdiwala's B-statistic was used to construct agreement charts, which provided a useful visual representation when comparing paired ordinal categorical data (27). When a perfect agreement is reached, rectangles are perfect-squared and the shaded squares are equal to the rectangles. Partial agreement could be identified by comparing the area of blackened squares to the area of the rectangles. Finally, observer bias could be identified on the basis of deviations from the 45° diagonal line.

Subgroup analysis was conducted by taking into account the orphan status and clinical indication (cancer vs. not cancer) according to AIFA decisions.

All statistical analysis was performed using R Statistical Software (version 4.0.4, vcd and irr packages).

## Results

### Sample selection

Of the 127 reports, a total of 25 were excluded for the following reasons: (a) for two medicinal products, the HAS reports were missing; (b) for two medicinal products, there were multiple ASMR scores given for the subpopulations analysis; and (c) for 18 medicinal products, there were multiple SMR scores given for the subpopulation analysis. Therefore, the analysis was conducted on 102 medicinal products for the same therapeutic indication. (Supplementary Figure S1)

### Description of the sample

As reported in Table 5, 38 (37.2%) were orphan drugs and 56 (54.9%) had a clinical indication for the treatment of cancer. The majority of the drugs had a non-curative indication (90, 88.2%), while almost half (56, 54.9%) were cancer treatments (or antineoplastic agents with L01 ATC code).

**Table 5:** Summary table on medical products included in the analysis.

	*N*	%
Total sample (*n*)	102	100
EMA approval		
CMA or EC	11	10.78
EC with AA	1	0.98
NR	72	70.59
NR with AA	18	17.65
Orphan status		
Yes	38	37.25
No	64	62.75
Cancer treatments		
Yes	56	54.90
No	46	45.10
Treatments		
Potentially curative	5	4.90
Non-curative	90	88.24
Prophylaxis	7	6.86
Indication		
Adult or paediatric/adolescent	22	21.57
Only adult	80	78.43
ATC		
L01—Antineoplastic agents	56	54.90
L04—Immunosuppressants	11	10.78
J05—Anti-infectives for systemic use	6	5.88
A16—Other alimentary tract and metabolism products	4	3.92
B01—Antithrombotic agents	3	2.94
B02—Antihaemorrhagics	3	2.94
N02—Analgesics	3	2.94
S01—Ophthalmologicals	3	2.94
M09—Other drugs for disorders of the musculoskeletal system	2	1.96
R05—Cough and cold preparations	2	1.96
R07—Other respiratory system products	2	1.96
Other ATCs	7	6.86

CMA, conditional market approval; EC, under exceptional circumstances; AA, accelerated assessment; NR, normal route; EMA, European Medicines Agency.

In the majority of cases (*n* = 72, 70.59%), the EMA approved the clinical indication following standard processes. For 11 (10.78%) medical products, EMA authorisation was conditional (CMA) (24) or recognised under exceptional circumstances (EC) (23). Only one case was an accelerated assessment (AA) (25) conducted under EC. In our analysis, we made two comparisons: normal route (NR) (*n* = 90) vs. no NR (*n* = 12) approvals and AA (*n* = 19) vs. no AA (*n* = 83).

### The assessments

The AIFA defined 38 (37.25%) drugs as fully innovative and 31 (30.4%) as conditionally innovative. In one case, it recognised a single medicinal product as having a maximum level of the TAV. The HAS, by contrast, awarded an ASMR I–II–III score to only 22 (21.6%) drugs and an ASMR IV score to 47 (46.1%) drugs (Table 6).

**Table 6:** AIFA and HAS judgments.

	AIFA		HAS	
	*N*	%	*n*	%
Innovativeness judgment				
Innovative	38	37.25	22	21.57
Conditional	31	30.39	47	46.08
Not innovative	33	32.35	33	32.35
Therapeutic added value				
Maximum	1	0.98	0	0.00
Important	33	32.35	4	3.92
Moderate	39	38.24	18	17.65
Poor	24	23.53	47	46.08
Absent	5	4.90	24	23.53
NE	0	0.00	9	8.82

The opinion expressed by the Italian agency for TAV was associated with the ASMR judgments expressed by the French agency. Thus, the opinions of the two agencies were associated as follows. For TAV: (a) Maximum TAV AIFA—ASMR I; (b) Important TAV AIFA—ASMR II; (c) Moderate TAV AIFA—ASMR III; (d) Poor TAV AIFA—ASMR IV; (e) Absent TAV AIFA—ASMR of V and ASMR NA. For innovation: (a) AIFA full innovativeness—ASMR I–III; (b) Conditional Innovation—ASMR IV; (c) Non-innovativeness—ASMR V and ASMR NA.

AIFA, Italian Medicines Agency; HAS, Haute Autorité de Santé; ASMR, Amélioration du Service Médical Rendu; TAV, therapeutic added value.

Table 6 shows that in Italy the judgment of full innovation is almost always associated with an important or maximum therapeutic added value. In just over half of the cases (*n* = 57, 55.9%), the AIFA and HAS assessed innovativeness in the same year (Supplementary Figure S2). The years of AIFA decisions on reimbursement in comparison with the years of assessment of innovativeness are reported in Supplementary Figure S2.

For a better interpretation of our results, the distribution of drugs with a cancer- or non-cancer-related indication was investigated in detail, as shown in Table 7. Taking as a reference the AIFA designation of innovativeness for cancer-related products, the innovative (39.29%) or conditional (35.71%) status is preferred, while for non-cancer-related products, the non-innovative status (41.30%) is a more common decision. In contrast, taking as a reference the HAS designation of innovativeness, for both groups, the conditional innovativeness is the most common decision (44.64% vs. 47.83%).

**Table 7:** Summary table on medicinal products included in the analysis focusing on the therapeutic indication.

Therapeutic indication	Cancer (*N* = 56)	No cancer (*N* = 46)
	Yes	No
Orphan status		
Yes	19 (33.93%)	19 (41.30%)
No	37 (66.70%)	27 (58.70%)
AIFA innovativeness judgment		
Innovative	22 (39.29%)	16 (34.78%)
Conditional	20 (35.71%)	11 (23.91%)
Not innovative	14 (25.00%)	19 (41.30%)
HAS ASMR score		
I–III	12 (21.43%)	10 (21.74%)
IV	25 (44.64%)	22 (47.83%)
V	19 (33.93%)	14 (30.43%)
EMA approval		
NR	51 (91.07%)	39 (84.78%)
Non-NR	5 (8.93%)	7 (15.22%)
AA	47 (83.93%)	36 (78.26%)
Non-AA	9 (16.07%)	10 (21.74%)

AA, accelerated assessment; AIFA, Italian Medicines Agency; ASMR, Amélioration du Service Médical Rendu; EMA, European Medicines Agency; HAS, Haute Autorité de Santé; NR, normal route.

### Agreement on the opinions issued by two agencies

The cross-analysis of the two national authorities assessments indicate a greater level of agreement for the AIFA's innovativeness recognition (equal to 63.7%) versus the HAS ASMR, lower instead for the AIFA TAV (equal to 14.7%). Table 8 reports the level of agreement between AIFA and HAS and particularly the statistical results: *p*-value for Fisher's exact test, raw agreement, kappa and weighted kappa. The information provided by Fisher's exact test does not lend itself to easy interpretation. The level and kind of agreement and disagreement among the two agencies are not clear.

**Table 8:** Level of agreement between AIFA and HAS.

		Fisher's exact test	Raw agreement	Kappa statistic		Weighted kappa			
	*n*	*p*-value	%	Unweighted	*p*-value	Equal	*p*-value	Squared	*p*-value
Assessment of TAV									
Overall sample	102	<0.001	14.7%	−0.066	0.121	0.13	<0.001	0.297	<0.001
* Subgroup analysis*	* *								
* * Clinical indication									
* * Cancer	56	0.011	8.9%	−0.118	0.0269	0.0525	0.168	0.198	<0.001
* * Not cancer	46	<0.001	21.7%	−0.001	0.993	0.231	<0.001	**0**.**422**	<0.001
* * Orphan status									
* * Orphan	38	0.017	15.8%	−0.035	0.59	0.116	0.031	0.237	<0.001
* * Not orphan	64	<0.001	14.1%	−0.094	0.0997	0.122	0.009	**0**.**303**	<0.001
* * AIFA innovativeness judgment									
* * Innovative	38	0.5412	21.1%	0.078	0.0674	0.0202	0.41	−0.036	0.354
* * Conditional	31	0.2909	3.2%	−0.023	0.196	0.0016	0.853	0.023	0.254
* * Not innovative	33	0.4603	18.2%	−0.124	0.117	−0.0377	0.535	0.030	0.751
* * EMA approval									
* * NR	90	<0.001	15.56%	−0.0669	0.152	0.146	<0.001	**0**.**350**	<0.001
* * Non-NR	12	0.4253	8.3%	−0.0645	0.501	0.0455	0.371	0.091	0.354
* * AA	19	0.3168	5.26%	−0.0789	0.283	0.0645	0.464	0.211	0.114
* * Non-AA	83	<0.001	16.87%	−0.053	0.265	0.148	<0.001	**0**.**310**	<0.001
Assessment of innovative status									
Overall sample	102	<0.001	63.7%	0.463	<0.001	0.547	<0.001	**0**.**638**	<0.001
*Subgroup analysis*	* *								
* * Clinical indication									
* * Cancer	56	<0.001	57.1%	0.362	<0.001	0.438	<0.001	0.522	<0.001
* * Not cancer	46	<0.001	71.7%	0.587	<0.001	0.679	<0.001	**0**.**778**	<0.001
* * Orphan status									
* * Orphan	38	<0.001	57.9%	0.38	<0.001	0.472	<0.001	0.578	<0.001
* * Not orphan	64	<0.001	67.2%	0.506	<0.001	0.581	<0.001	**0**.**661**	<0.001
* * EMA approval									
* * NR	90	<0.001	63.3%	0.456	<0.001	0.546	<0.001	**0**.**645**	<0.001
* * Non-NR	12	0.2309	66.7%	**0**.**505**	0.0102	0.552	0.013	0.596	**0**.**0304**
* * AA	19	0.4083	42.1%	0.167	0.196	0.249	0.126	0.334	0.118
* * Non-AA	83	<0.001	68.7%	0.527	<0.001	0.611	<0.001	**0**.**705**	<0.001

Unweighted kappa considered all disagreements equally; weighted kappa assigned a different weight to disagreements according to the magnitude of the discrepancy. Bold values indicate agreement on the opinions issued by two agencies.

AA, accelerated assessment; AIFA, Italian Medicines Agency; ASMR, Amélioration du Service Médical Rendu; EMA, European Medicines Agency; HAS, Haute Autorité de Santé; NR, normal route; TAV, therapeutic added value.

However, according to the unweighted statistical kappa, the agreement for the recognition of innovativeness is moderate (*k* = 0.463, *p*-value = <0.0001) (Table 8).

The results of the analysis of the comparison between the TAV according to the AIFA and the ASMR according to the HAS show a lack of agreement between the two agencies (*k* < 0, *p*-value = 0.121); however, according to the weighted kappa analysis, a partial agreement emerges (weights squared *k* = 0.297, *p*-value <0.0001).

From the analysis of the “*Clinical indication*” subgroup, a moderate agreement emerges between the two agencies (k-squared = 0.422, *p* < 0.001) only for non-cancer. Moreover, for the same subgroup, the AIFA kappa statistic on innovativeness was the highest of all analyses and demonstrated good agreement (k-squared = 0.788, *p* < 0.001).

A similar trend, although not as marked, emerged for the non-orphans in the “*Orphan status*” subgroup (k = 0.661, *p* < 0.001). The analysis of the “*Innovation rating*” subgroup shows poor agreement in the evaluation of the AIFA TAV vs. ASMR (all the kappa statistics are <0.1 for each innovation rating group).

By comparing the data obtained from the analysis for the “*EMA approval*” subgroup, a better agreement emerges between the judgment of AIFA's innovativeness rather than the judgment of AIFA's TAV towards the ASMR of the HAS. In addition, the result of the agreement for the innovation of AIFA vs. ASMR of the HAS on the total sample is also confirmed for the subcategories analysed. In fact, for the NR and non-AA groups, the agreements are good (k-squared = 0.645, *p* < 0.001 and k-squared = 0.705, *p* < 0.001 respectively).

**Figure 1:**
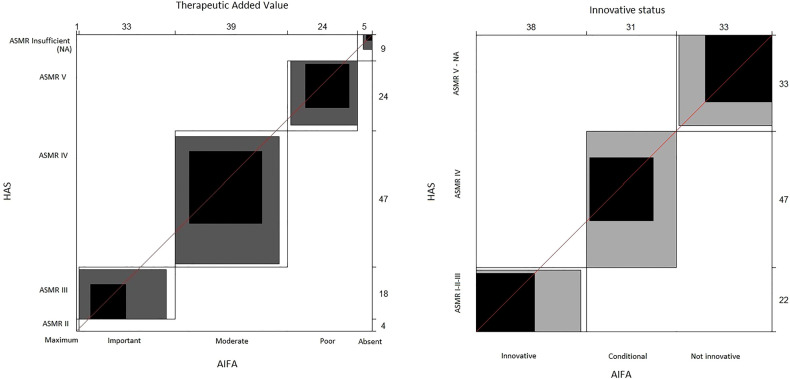
Agreement charts. The unweighted kappa treats all discrepancies equally; the weighted kappa assigns a different weight to discrepancies based on the magnitude of the discrepancy. When a perfect agreement is reached, rectangles are perfect-squared and the shaded squares are equal to the rectangles. Partial agreement is identified by comparing the area of blackened squares to the area of the rectangles. Observer bias can identify deviations from the 45° diagonal line. AIFA, Italian Medicines Agency; ASMR, *Amélioration du Service Médical Rendu*; HAS, Haute Autorité de Santé.

The agreement graphs (Figure 1) confirm the lack of perfect agreement between the two agencies. It seems evident that the AIFA tends to assign the TAV a higher ranking than the ASMR of the HAS (Table 6). In fact, whereas the AIFA tends to recognise the TAV as *important*, the ASMR of the HAS recognises it as being of *moderate* importance. In addition, although both agencies have a bias towards the conditional innovativeness, it is shown by the different rectangles (Figure 1) that the agreement is only partial.

## Discussion

This article aims to investigate how the evaluation of a medicine is performed by the Italian (AIFA) and the French agencies (HAS) bearing in mind the National Value Framework present in the two countries. Both are self-governing agencies and adopt similar criteria to assess the clinical benefit of medicinal products for pricing and reimbursement decisions. The results of the analyses, based on agreement statistics, show how these criteria affect the overall judgment differently.

### AIFA TAV vs. HAS ASMR

Considering what has been described in the previous paragraphs, the opinion expressed by the AIFA on the TAV was associated with opinions regarding improvement in actual benefit (ASMR) expressed by the HAS. The assumption was based on the similar wording and ranking of the TAV and ASMR.

Specifically, the TAV represents the extent of the clinical benefit provided by the medicinal product on outcomes recognised and validated as clinically relevant. The TAV is divided into five levels (11, 12), as reported in Table 2. The ASMR, on the other hand, considers the added therapeutic value that the medicinal product brings compared with therapeutic alternatives for the same therapeutic indication and the improvement it brings. Thus, the ASMR judgment determines whether the drug improves the clinical condition of patients compared with existing therapies (17). Even the ASMR, like the AIFA TAV, is declined through a score based on a scale ranging from 1 to 5 (17, 19), as reported in Table 2.

Although the wording of *added* or *improvement* in clinical benefit (the TAV or ASMR) between the two agencies seems similar, the analysis shows a lack of agreement between the two HTA authorities on the total sample of 102 medicinal products (Table 8). In fact, a comparison of TAV judgments shows how the AIFA gives more Important or Moderate judgments, while the HAS tends to assign Low or even Absent ASMR judgments of value (Table 6). The explanations are related to the different methodologies. Otherwise, AIFA's TAV assessment needs to be integrated with the other two criteria for an overall assessment (innovation). The resulting lack of agreement between the TAV and the ASMR shows how the similar wording of value is not suitable for analysing the differences in opinions expressed by the two agencies.

An analysis of agreements for the TAV by subgroups/subcategories also confirms this disagreement observed in the total sample.

In the subcategory “*Clinical indication*” for cancer drugs, there is no agreement (kappa score <0.2) (Table 8). In addition, by stratifying the cancer drugs by the other variables considered in the analysis (Table 7), it is highlighted that these products mostly have no orphan designation and have been approved with a standard EMA regulatory procedure (normal route). On this point, further examinations are needed (clinical study design, trial efficacy and safety endpoints, comparator control arm, etc) to understand the differences between the two subgroups. In this regard, it is useful to remember that the AIFA considers OS to be the gold standard. The lack of OS data must be adequately justified and, in relation to the tumour type and therapeutic setting, progression-free survival (PFS), disease-free survival, the duration of complete response, or other surrogate outcomes whose predictive value of clinical benefit is also recognised on the basis of the entity of the effect can be considered (11, 12). The HAS, on the other hand, in expressing its judgment on the ASMR, not only considers OS and PFS but also focuses on the safety profile of the drug, the quality of the studies presented, and the outcomes of the endpoints (20). This particularity is evident: more than half of the cancer drugs analysed include expressly detailed safety components in the ASMR assessments vs. that of the AIFA with only one-third of the cases.

• The AIFA, in its assessment on brentuximab vedotin for the treatment of cutaneous T-cell lymphoma, considers the efficacy data obtained in terms of sustained response and PFS. Moreover, the agency stresses that clinical evidence is limited to the subpopulation of patients with mycosis fungoides and primary cutaneous anaplastic large cell lymphoma (28). The HAS, in its evaluation, adds further elements to the safety profile associated with significant neurotoxicity and the lack of OS data (29). These additional elements lead the French agency to assign an ASMR IV with respect to Moderate AIFA TAV ranking. Overall, the Italian Agency assigns a conditional innovation status and incorporates the elements contained in the ASMR judgment such as the clinical benefit and the quality of the randomised clinical trial (24). Furthermore, as is the case for innovative drugs (30), after the pricing and reimbursement process decision is taken, brentuximab is monitored through the AIFA appropriateness registry (31).
• Another example is reported from an analysis of the evaluation of the combination BRAF and MEK inhibitor (dabrafenib/trametinib) for stage III melanoma with BRAF600 mutation positive. The AIFA considers the advantage in terms of PFS and OS compared with placebo by assigning the Important TAV score (32), thus recognising full innovation status (and also implementing the AIFA appropriateness registry) (33). The HAS, on the other hand, recognises the advantage in terms of PFS like the Italian authority but considers the OS data insufficient and additionally includes the safety profile in the evaluation, assigning an ASMR III (Moderate) (31).

This difference in approach can also be observed when both agencies assign the comparable TAV/ASMR ranking.

• For nivolumab (fully innovative for AIFA) indicated for the adjuvant treatment of adults with melanoma with involvement of lymph nodes or metastatic disease who have undergone complete resection, both agencies assigned a Moderate score (the HAS corresponding to ASMR III), but the French agency, in assigning the ASMR rating, considers, in addition to relapse-free survival and OS, the frequency of adverse events, a criterion not explicitly considered by the AIFA in its evaluation (34, 35). Here, too, the AIFA sets up an *ad hoc* appropriateness registry (31).

In the subcategory “*Orphan status*,” it can be observed that the agreement among orphan drugs is very low (kappa score <0.2), but there is a partial agreement (kappa score = 0.3) for non-orphan drugs. By stratifying the analysis for non-orphan drugs, it is seen that these are mostly of cancer indication and are approved with the NR of the EMA. An analysis of the reports reveals a diversity of elements considered in the evaluation of the value by the two agencies.

• For midostaurin with indication for the treatment of acute myeloid leukaemia, the AIFA considers only OS in its evaluation, while the HAS together with OS also considers the degree of uncertainty of the data obtained from the trial in the evaluation (36). However, it is necessary to emphasise that the AIFA also considers the quality of the evidence, and that the judgment is separate from the TAV judgment, which is not the case for the HAS. In addition, midostaurin has an AIFA appropriateness registry (31). The separation of data by the AIFA with respect to the incorporation of data in a single judgment by the French agency explains the lack of agreement between the TAV scores and the ASMR scores (37).
• Another example is voretigene neparvovec for the treatment of vision loss due to retinal dystrophy, for which both agencies gave the same “important” judgment (ASMR II). In the evaluation, both agencies considered the primary and secondary endpoints and the absence of long-term data; however, unlike the AIFA (38), the HAS also considers the absence of data on quality of life (39). The AIFA recognises the full innovation (as well as the appropriateness product-based registry) (31), while it is confirmed that the judgment of innovativeness incorporates the inherent elements of the ASMR evaluation of the HAS, such as the high unmet clinical need and the low quality of evidence given by the distortion of the primary endpoint and by the imprecision of the statistical analysis.

An analysis of the subcategory “*Judgment of innovativeness*” reveals a lack of agreement (kappa score <0.2), while for the subcategory “*EMA approval*,” it highlights a partial agreement (kappa score = 0.3) for medicines approved with the normal route and without accelerated assessment. When carrying out a stratified analysis for this last subgroup, it is clear that most of the drugs have no orphan design. Furthermore, the reports reveal the lack of alignment between the two agencies in the elements considered for the evaluation of the therapeutic added value.

• An example is given by pembrolizumab with indication for the treatment of renal cell carcinoma, for which the AIFA considers as decisive elements the PFS, the OS, and the reduction of the risk of death compared with its comparator, while the HAS, in addition to the factors considered by the Italian agency in its assessment, also takes into account toxicity and the lack of data on quality of life (40, 41). Furthermore, in this case, there is also an *ad hoc* registry for this therapeutic indication (out of eight for this product) (31).

A similar result was obtained by Rodwin et al (42), who assessed medicines with oncology indication introduced in France between 2004 and 2017 by examining the relationship between the price of the medicines and their added therapeutic benefit as defined by the HAS through the ASMR and the European Society for Medical Oncology (ESMO) through the Magnitude of Clinical Benefit Scale (MCBS). The results of the study reveal a statistically significant low correlation between the ASMR or MCBS judgment and the price of drugs. The study also shows that the ASMR and ESMO judgments on added therapeutic value are divergent, which is probably due to their differing methodologies. Indeed, the ESMO generates a score for each clinical study, whereas ASMR assessments include all available clinical studies and take into account the choice of comparators, their application in clinical practice, and new data or an interpretation of past studies. Furthermore, they sometimes choose different comparators and adopt differing approaches to how they evaluate the drug efficacy, toxicity, and impact on quality of life (42). A similar analysis was reported by Li et al., who looked at the ASCO framework and thus found further inconsistency among the appraisals (43).

### AIFA innovativeness recognition vs. HAS ASMR

The judgment of innovativeness conferred by the AIFA is based on the 1525/2017 Deliberation (12), which introduces a multidimensional approach based on the combination of three criteria: unmet need, TAV, and quality of evidence. The French agency HAS, on the other hand, does not explicitly form a judgment on innovativeness, meaning that to conduct an evaluation of the agreement between the two agencies, the ASMR judgments were grouped as follows: (a) AIFA full innovativeness—ASMR score I, II, III; (b) conditional innovation—ASMR IV; (c) non-innovativeness—ASMR V (19, 20).

The results indicate a good agreement of 62.7% between the two agencies for the innovative status conferred on the total sample of 102 medicinal products (Table 8). An analysis of the agreement for innovation by subcategories confirms this agreement between the two agencies, with the concordance values being similar (Table 8).

In the subcategory “*Orphan status*,” it is possible to see that there is a modest agreement between orphan drugs (kappa score = 0.57) and a good agreement for non-orphan drugs (kappa score = 0.66).

As regards the last subcategory, “*EMA approval*” (22–25), there is a significant agreement between the two agencies for medicines approved with the NR and without AA. It can be hypothesised that the agreement obtained for medicines approved with the NR and without AA may be due to a lower clinical uncertainty of the data provided in the dossier. In most cases, the medicines that access the NR are non-orphan and/or non-cancer medicines, thus allowing for a greater completeness of the data and a lower clinical uncertainty. As mentioned in the previous paragraph, further in-depth analysis is required. The clinical uncertainty arises from the design of the study, the population or the choice of comparators, and the primary and secondary endpoints (44). It is important to note that the uncertainty around the effects of the treatments is inevitable: when a technology is first tested in humans, the effects can be predicted but cannot be known. Any evidence generated may have large confidence intervals or an apparent benefit related to some outcomes or patient subgroups but not to others (45). Furthermore, the comparator in the study may not reflect the standard of care in the country where the HTA takes place, and therefore, the relative efficacy may be unclear. Finally, even the study design can create an additional level of uncertainty, as small and/or single-arm studies make it difficult to estimate clinical efficacy (46).

For a better interpretation of the results, the distribution of drugs with clinical indication “*Cancer/non-cancer*” was studied in detail (Table 7). Taking as a reference the designation of innovativeness by the AIFA, the cancer drugs were mainly assigned full (39.29%) or conditional (35.71%) innovativeness. Non-innovative status, by contrast, was mainly assigned to non-oncological medicines (41.30%). With regard to the comparison of the innovativeness of the AIFA with the ASMR judgments of the HAS, for both the groups of cancer and non-cancer drugs, it was primarily conditioned innovativeness that was recognised—ASMR IV (44.64% vs. 47.83%). In addition, the similarity between the judgments expressed by the two agencies can also be observed for the full innovativeness—ASMR I–II–III score (21.43% vs. 21.74%)—and for the non-recognition of innovativeness—ASMR V and ASMR NA score (33.93% vs. 30.43%). The similarity trend between the opinions expressed by the two agencies can be explained by the fact that the AIFA's judgment of innovativeness incorporates the elements used by the HAS in the ASMR judgment.

• An example is patisiran with indication of the treatment of hereditary transthyretin amyloidosis, for which the AIFA reported that the therapeutic need is considered (the first AIFA criterion of innovation framework) for the treatment of stage I and II polyneuropathy, the achievement of primary and secondary endpoints, and finally moderate quality of evidence (47). The same considerations were made by the HAS in expressing the ASMR judgment (48).
• Another example is cerliponase alfa, which is used for the treatment of type 2 neuronal ceroid lipofuscinosis (CLN2), in that both agencies consider the therapeutic need and the clinical trial data as clinically relevant as well as taking into account the limitations arising from the observational study (49, 50).

Moreover, in these two cases, the Italian authority has established appropriateness product-based registries (31).

With regard to AIFA's judgment of innovativeness compared with the ASMR score, there is a statistically significant percentage of agreement between the two agencies, which is probably due to the fact that the AIFA in the innovation requirements includes two other criteria in addition to the TAV (unmet need and quality of evidence), thus approaching the standards for assessing the improvement of clinical benefit by the HAS or ASMR. Furthermore, it is possible to say that the highest levels of agreement both on the TAV and on the status of innovativeness were obtained for non-cancer and non-orphan medicinal products with EMA NR (standard) approval.

### Limitations, reflections, and further developments

Our results show how the HAS ASMR assessments contain almost all the elements used by the AIFA in the innovation reports, which is why there is greater agreement when these are compared with each other. To ensure a correct analysis, it will be necessary to extract and analyse in detail the AIFA/HAS assessment report (info on clinical trial, endpoints, comparators, tolerability, HRQoL, long-term data, etc.) in order to achieve greater clarity on the evaluation criteria used by the AIFA and HAS.

As represented in Table 2 and in the Data comparison section, it is clear that although the scores for the AIFA and HAS are in the same table rows, they are not aligned to be at the same level and also not to be at the same distances (it is not a fixed shift: AIFA TAV Absent and HAS ASMR V are very close if not the same, where this is not the case for AIFA TAV Moderate and HAS ASMR III). Furthermore, innovativeness is not scored independently by the HAS, so is a simple split in the ASMR score. It is clear that a detailed analysis of the innovativeness contents for the AIFA and ASMR for the HAS and also other supplementary features to the Italian and French decisions could clarify various aspects that do not emerge in this analysis, including the acceptability of surrogate endpoints and the typology of the designs of clinical studies.

One of the most important limitations is AIFA's ASMR rating criteria. As also reported in the previous paragraphs, our approach is based on the figures and definitions of the criteria used by the two agencies. Given the difficulty of rating, and recognising it as a limitation of our study, we report the numbers of our sample that justify it in Supplementary Table S2. The criticality is particularly for cases with HAS ASMR IV assignment that are practically distributed over the three groups. Other grouping systems could be explored; for example, AIFA full and conditional innovative status could be grouped together and compared with HAS ASMR I–IV.

Our analysis did not go into the details of reimbursement in the two countries: almost all products were reimbursed both in Italy (95.1%) and in France (90.2%). To this, however, we must add the model of managed entry agreements (MEAs) and registries that link the pricing and reimbursement decision and the level of uncertainty of the medicinal product for a specific therapeutic indication. Perhaps, this could partly explain AIFA's generosity in assigning higher TAV rankings: innovative drugs are generally subject to AIFA monitoring through registries. The AIFA registries (30) are intended to verify the appropriateness and, until a few years ago (2017) (with the exception of one outcome-based agreement for lumasiran in 2022), used the MEA to manage clinical/economic uncertainty. National data generated from a product-based registry (only for patients treated), therefore, aim to control the pharmaceutical expenditure of innovative drugs, replicating the eligible criteria of clinical trials in the post-marketing phase. The AIFA and HAS differ in this regard, in the sense that the former tries to manage uncertainty through monitoring registries and entry agreements (30, 51), while the latter uses new evidence generated by clinical practice in order to re-evaluate the ASMR judgment (20).

Nicod et al. (52) developed a conceptual framework that integrates the factors explaining the HTA decisions and drug reimbursement by exploring their relationship and assessing whether they are congruent, complementary, or discrepant. France was one of the countries selected for the analysis. Among the lessons learnt, the authors report that while cross-country differences in reimbursement recommendations are legitimate as they reflect local decision-making, they may also be a consequence of differences in the methodologies, timelines, evidence considered, and review of evidence across settings, and it is, therefore, important to identify and understand these (52).

The results of our analysis are also aligned with the scientific literature.

Similar observations, meanwhile, were reached by Angelis et al. (7), who carried out a critical review of the evaluation methods of new medicines in eight European countries and their respective HTA agencies (France, Germany, Sweden, England, Netherlands, Italy, Poland, and Spain). The study highlights a number of significant similarities but also notable differences in evaluation practices, processes, and policies in the countries under study. The authors conclude by saying that the differences found are due to the different national priorities between the countries but are also due to the different methodological processes and frameworks adopted for the collection of decision-makers' preferences. Currently, all these decisions are subject to the discretion of decision-makers, but in most cases, they are presented in a non-transparent way (7).

Finally, the temporal range of our study is also limited. Our data extraction does not include those assessed before 2017 with the old innovation model because the AIFA innovation assessment reports were not transparent (53). This limited our comparison analysis with the HAS reports. However, the literature reports that even the old AIFA innovation framework (53) vs. HAS ASMR still presented some discrepancies (54).

Our results are also in line with those of another article (55), which compares the agreement of cancer drug reimbursement decisions in nine European countries. Although there is no comparison between France and Italy (Italy is not among the selected countries), this study reported a medium agreement among decisions adopted from 2002 to 2014 by Belgium, Germany, France, Spain, Sweden, Portugal, Poland, England, and Scotland. The adopted methodology is similar, being based on the Cohen's kappa (55). In addition, our analysis investigated partial agreement, considered a more recent timeframe (July 2017–September 2021), and is not limited to cancer drugs. We, therefore, focused not on final decisions (acceptance, restriction, or rejection) but on key elements that influence the reimbursement decisions: assessment of the AIFA TAV/innovative status and HAS ASMR ranking.

Despite the integrations considered in our analysis for the Cohen k statistics, it should be acknowledged that such an approach had some limitations (56). First of all, a unique scale to interpret results was not available, and those that were available referred to clinical contexts. We specified the adopted scale (26). Also, Cohen's statistics could be influenced by asymmetric tables. For this reason, as reported above, we also considered partial agreement with the weighted kappas. Therefore, our analysis, even if it could benefit from further improvements, attempted to be as complete and transparent as possible in terms of the current agreement between the assessments of Italy and France.

## Conclusions

An analysis of the AIFA reports shows that in Italy, the judgment of full innovation is almost always associated with an important or maximum therapeutic added value. As for the comparison with France, the results show differences between the AIFA and the HAS assessments. Bearing in mind the ASMR expressed by the HAS, its comparison with the AIFA on the TAV score demonstrates a lack of agreement. It is different if this comparison is towards the judgment of innovativeness where the presence of agreement is highlighted.

In light of the results obtained, the AIFA would seem significantly more “generous.” This would also explain the greater agreement obtained between the AIFA innovation rating and the ASMR score, as the AIFA's innovation rating incorporates the elements used by the HAS. Furthermore, separating the elements of the individual reports and extracting the data of both agencies would allow for greater clarity regarding the evaluation criteria used by the AIFA and HAS.

These results underline the importance of implementing procedures that are characterised by a greater transparency in terms of the value definition criteria used by HTA organisations as well as the importance of ensuring that European health technology assessments are as standardised and harmonised as possible with the expected effect in terms of pricing and reimbursement of medicines, access to treatments, etc. The new EU regulation of the HTA (although now voluntarily in the early years in the preparatory phase) could be a test case for national authorities.

## References

[1] Regulation (EU) 2021/2282 on health technology assessment and amending Directive 2011/24/EU. Available at: https://ec.europa.eu/health/health-technology-assessment/regulation-health-technology-assessment_en (Accessed April 5, 2022).

[2] EUnetHTA. (2022). Available at: https://www.eunethta.eu/ (Accessed April 5, 2022).

[3] BeNeLuxA website. Available at: https://beneluxa.org/ (Accessed April 5, 2022).

[4] Nordic Pharmaceutical Forum and FiNoSe website. (2022). Available at: https://amgros.dk/en/about-amgros/cooperation-partners/international-cooperation/ (Accessed April 5, 2022).

[5] Visegrad—“Fair & Affordable Pricing” (FAAP) website. (2022). Available at: https://www.gov.pl/web/zdrowie/faap (Accessed April 5, 2022).

[6] Testori Coggi P. Valletta Declaration (Internet). Facing the challenges: equity, sustainability and access. (2018). Available at: https://www.infarmed.pt/documents/15786/2835945/Paola_Testori_Coggi.pdf/2388762b-7506-4a78-9533-7422ea480c55 (Accessed April 5 2022).

[7] Angelis A Lange A Kanavos P. Using health technology assessment to assess the value of new medicines: results of a systematic review and expert consultation across eight European countries. Eur J Heal Econ. (2018) 19(1):123–52. 10.1007/s10198-017-0871-0 PMC5773640 28303438

[8] Allen N Liberti L Walker SR Salek S. A comparison of reimbursement recommendations by European HTA agencies: is there opportunity for further alignment? Front Pharmacol. (2017) 8:384. 10.3389/fphar.2017.00384 PMC5491965 28713265

[9] AIFA website. Available at: www.aifa.gov.it (Accessed April 5, 2022).

[10] Gazzetta Ufficiale. Italian Law 11/12/2016 N. 232 Comma 404. (2016). Available at: http://www.gazzettaufficiale.it/eli/id/2016/12/21/16G00242/sg (Accessed April 5, 2022).

[11] Gazzetta Ufficiale. Deliberation 2017/519 Innovativeness Recognition Scheme, New Criteria: Website. (2017). Available at: http://www.gazzettaufficiale.it/eli/id/2017/04/05/17A02486/sg;jsessionid=qvKOr+GBz85IVCTpcGUakg__.ntc-as5-guri2b (Accessed April 5, 2022).

[12] Gazzetta Ufficiale. Update on Innovativeness Recognition Scheme, New Criteria (AIFA Deliberation N. 1535/2017). (2017). Available at: http://www.gazzettaufficiale.it/eli/id/2017/09/18/17A06376/sg;jsessionid=PihxCVLIHx-9P+ZUonsYUA__.ntc-as3-guri2a (Accessed April 5, 2022).

[13] Fortinguerra F Perna S Marini R Dell'Utri A Trapanese M Trotta F The assessment of the innovativeness of a new medicine in Italy. Front Med. (2021) 8:793640. 10.3389/fmed.2021.793640 PMC8692651 34957163

[14] Fortinguerra F Tafuri G Trotta F Addis A. Using GRADE methodology to assess innovation of new medicinal products in Italy. Br J Clin Pharmacol. (2020) 86(1):93–105. 10.1111/bcp.14138 31656055 PMC6983505

[15] Galeone C Bruzzi P Jommi C. Key drivers of innovativeness appraisal for medicines: the Italian experience after the adoption of the new ranking system. BMJ Open. (2021) 11(1):e041259. 10.1136/bmjopen-2020-041259 33441356 PMC7812109

[16] GRADE Working Group. Grading quality of evidence and strength of recommendations. Br Med J. (2004) 328:1490. 10.1136/bmj.328.7454.1490 15205295 PMC428525

[17] Safon M. O Suhard V. La politique du médicament en France: aspects historiques et réglementaires. (2021). Available at: https://www.irdes.fr/documentation/syntheses/historique-de-la-politique-du-medicament-en-france.pdf (Accessed April 5 2022).

[18] CEPS. *Comité Economique des Produits de Santé* website. (2022). Available at: https://solidarites-sante.gouv.fr/ministere/acteurs/instances-rattachees/article/ceps-comite-economique-des-produits-de-sante (Accessed April 5, 2022).

[19] Sermet C. La prise en compte de l’innovation thérapeutique dans les politiques de prix et de remboursement des médicaments: une approche internationale. Revue Française des Affaires Sociales, n° 3/4 (2007).

[20] HAS. (2021). Available at: https://www.has-sante.fr/jcms/c_1002212/fr/missions-de-la-has (Accessed April 5, 2022).

[21] AIFA. Innovation Assessment Report Website. (2021). Available at: https://www.aifa.gov.it/en/web/guest/farmaci-innovativi Updated June 23, 2020 (Accessed April 5, 2022).

[22] EMA. Orphan Designation Website. (2022). Available at: https://www.ema.europa.eu/en/human-regulatory/marketing-authorisation/orphan-designation-marketing-authorisation (Accessed April 5, 2022).

[23] EMA. Exceptional Circumstances Website. (2022). Available at: https://www.ema.europa.eu/en/glossary/exceptional-circumstances (Accessed April 5, 2022).

[24] EMA. Conditional Marketing Authorisation Website. (2022). Available at: https://www.ema.europa.eu/en/human-regulatory/marketing-authorisation/conditional-marketing-authorisation (Accessed April 5, 2022).

[25] EMA. Accelerate Assessment Website. (2022). Available at: https://www.ema.europa.eu/en/human-regulatory/marketing-authorisation/accelerated-assessment (Accessed April 5, 2022).

[26] Altman DG. Practical statistics for medical research. London: Chapman and Hall (1991).

[27] Bangdiwala SI Shankar V. The agreement chart. BMC Med Res Methodol. (2013) 13:97. 10.1186/1471-2288-13-9 23890315 PMC3733724

[28] AIFA. Adcetris CTCL Innovation Assessment Report. (2018). Available at: https://www.aifa.gov.it/documents/20142/1100047/ADCETRIS_INNOV_13062_1.0.pdf (Accessed April 5, 2022).

[29] HAS. Adcentris ASMR. (2019). Available at: https://www.has-sante.fr/jcms/c_2963333/fr/adcetris-brentuximab-vedotin%0A (Accessed April 5, 2022).

[30] Xoxi E Facey KM Cicchetti A. The evolution of AIFA registries to support managed entry agreements for orphan medicinal products in Italy. Front Pharmacol. (2021) 12:699466. 10.3389/fphar.2021.699466 34456724 PMC8386173

[31] AIFA. Update List of Web-Based Registries and Therapeutic Plan Website. (2022). Available at: https://www.aifa.gov.it/en/web/guest/registri-e-piani-terapeutici1 (Updated August 5, 2020. Accessed April 5, 2022).

[32] AIFA. Tafinlar Innovation Assessment. (2019). Available at: https://www.aifa.gov.it/documents/20142/1100047/MEKINIST_14016_TAFINLAR_14017_melanoma+adiuvante_INNOV_1.0.pdf (Accessed April 5, 2022).

[33] HAS. Mekinist-tafinlar ASMR. (2019). Available at: https://www.has-sante.fr/jcms/c_2911595/fr/mekinist-tafinlar-trametinib/-dabrafenib (Accessed April 5, 2022).

[34] AIFA. Opdivo Innovation Assessment Report. (2019). Available at: https://www.aifa.gov.it/documents/20142/1100047/OPDIVO_13880_MELANOMA_ADIUV_INNOV_v1.0.pdf (Accessed April 5, 2022).

[35] HAS. Opdivo. (2019). Available from: https://www.has-sante.fr/jcms/c_2897014/en/opdivo-melanome-nivolumab (Accesses April 5, 2022).

[36] AIFA. Rydapt Innovation Assessment Report. (2017). Available at: https://www.aifa.gov.it/sites/default/files/Rydapt_SM.pdf (Accessed April 5, 2022).

[37] HAS. Rydapt ASMR. (2018). Available at: https://www.has-sante.fr/jcms/c_2862069/fr/rydapt-midostaurine-inhibiteur-de-tyrosine-kinase (Accessed April 5, 2022).

[38] AIFA. Luxturna Innovation Assessment Report. (2020). Available at: https://www.aifa.gov.it/documents/20142/1308577/105_Luxturna_14183_scheda_innovativita_GRADE.pdf (Accessed April 5, 2022).

[39] HAS. Luxturna ASMR. (2019). Available at: https://www.has-sante.fr/jcms/c_2964759/fr/luxturna-voretigene-neparvovec (Accessed April 5, 2022).

[40] AIFA. Keytruda Innovation Assessment Report. (2020). Available at: https://www.aifa.gov.it/documents/20142/1308577/103_Keytruda_14796_scheda_innovativita_GRADE.pdf (Accessed April 5, 2022).

[41] HAS. Keytruda ASMR. (2020). Available at: https://www.has-sante.fr/jcms/p_3184677/fr/keytruda-cancer-du-rein-pembrolizumab (Accessed April 5, 2022).

[42] Rodwin MA Mancini J Duran S Jalbert AC Viens P Maraninchi D The use of “added benefit” to determine the price of new anti-cancer drugs in France, 2004–2017. Eur J Cancer. (2021) 145:11–8. 10.1016/j.ejca.2020.11.031 33412466

[43] Li J Vivot A Alter L Durand-Zaleski I. Appraisal of cancer drugs: a comparison of the French health technology assessment with value frameworks of two oncology societies. Expert Rev Pharmacoecon Outcomes Res. (2020) 20(4):405–9. 10.1080/14737167.2019.1635458 31240965

[44] Trowman R Powers A Ollendorf DA. Considering and communicating uncertainty in health technology assessment. Int J Technol Assess Health Care. (2021) 37(1):e74. 10.1017/S0266462321000453 34261561

[45] Sandercock PA. Short history of confidence intervals: or, don’t ask “does the treatment work?” but “how sure are you that it works?”. Stroke. (2015) 46(8):e184–7. 10.1161/STROKEAHA.115.007750 26106115

[46] Vreman RA Naci H Goettsch WG Mantel-Teeuwisse AK Schneeweiss SG Leufkens HGM Decision making under uncertainty: comparing regulatory and health technology assessment reviews of medicines in the United States and Europe. Clin Pharmacol Ther. (2020) 108(2):350–7. 10.1002/cpt.1835 32236959 PMC7484915

[47] AIFA. Onpattro Innovation Assessment Report. (2018). Available at: https://www.aifa.gov.it/documents/20142/1184740/ONPATTRO_13833_INNOV._v.1.0.pdf (Accessed April 5, 2022).

[48] HAS. Onpattro. (2019). Available from: https://www.has-sante.fr/jcms/c_2912140/fr/onpattro-patisiran (Accessed April 5, 2022).

[49] AIFA. Brineura Innovation Assessment Report. (2018). Available at: https://www.aifa.gov.it/documents/20142/1184740/BRINEURA_12650_INNOV._+v.1.0.pdf (Accessed April 5, 2022).

[50] HAS. Brineura ASMR. (2018). Available at: https://www.has-sante.fr/jcms/c_2859880/fr/brineura-cerliponase-alfa-classe-simple (Accessed April 5, 2022).

[51] Cicchetti A Coretti S Iacopino V Montilla S Xoxi E Pani L Italy post-marketing successful strategies to manage pharmaceutical innovation. Success stories from 60 countries. In: Jeffrey B Mannion R Yukihiro M Shekelle P, editors. Health systems improvement across the globe. London: Taylor & Francis. (2017). p. 192–7. Available at: https://m.ebrary.net/99655/health/health_systems_improvement_across_the_globe_success_stories_from_60_countries (Accessed April 5, 2022).

[52] Nicod E Maynou L Visintin E Cairns J. Why do health technology assessment drug reimbursement recommendations differ between countries? A parallel convergent mixed methods study. Health Econ Policy Law. (2020) 15(3):386–402. 10.1017/S1744133119000239 31488229

[53] AIFA. Italian Medicines Agency WGoID. Criteria for Ranking Therapeutic Innovation of New Drugs and Elements for Supplementing the Dossier for Admission to the Reimbursement System. (2007). Available at: http://www.agenziafarmaco.gov.it/allegati/integral_document.pdf (Accessed April 5, 2022).

[54] Solaman DA Chandler T Wright A. Innovation ranking in France and Italy: differences and their impact on pricing and reimbursement processes. Value Health. (2015) 18(7):A560. 10.1016/j.jval.2015.09.1822

[55] Maynou L Cairns J. Disagreement on cancer drug decisions in Europe. Int J Technol Assess Health Care. (2020) 36(3):232–8. 10.1017/S026646232000032X 32538341

[56] McHugh ML. Interrater reliability: the kappa statistic. Biochem Med. (2012) 22(3):276–82. 10.11613/BM.2012.031 PMC3900052 23092060

