# Supplementary material for: Value assessment of medicinal products by the Italian Medicines Agency (AIFA) and French National Authority for Health (HAS): Similarities and discrepancies
Source: Front Med Technol. 2022 Sep 5;4:917151. doi: 10.3389/fmedt.2022.917151 (PMC9483157; doi:10.3389/fmedt.2022.917151)
Supplement: Supplementary file 1 [file Data_Sheet_1.pdf]

# 1 Supplementary materials

**Supplementary Figure 1. Database**

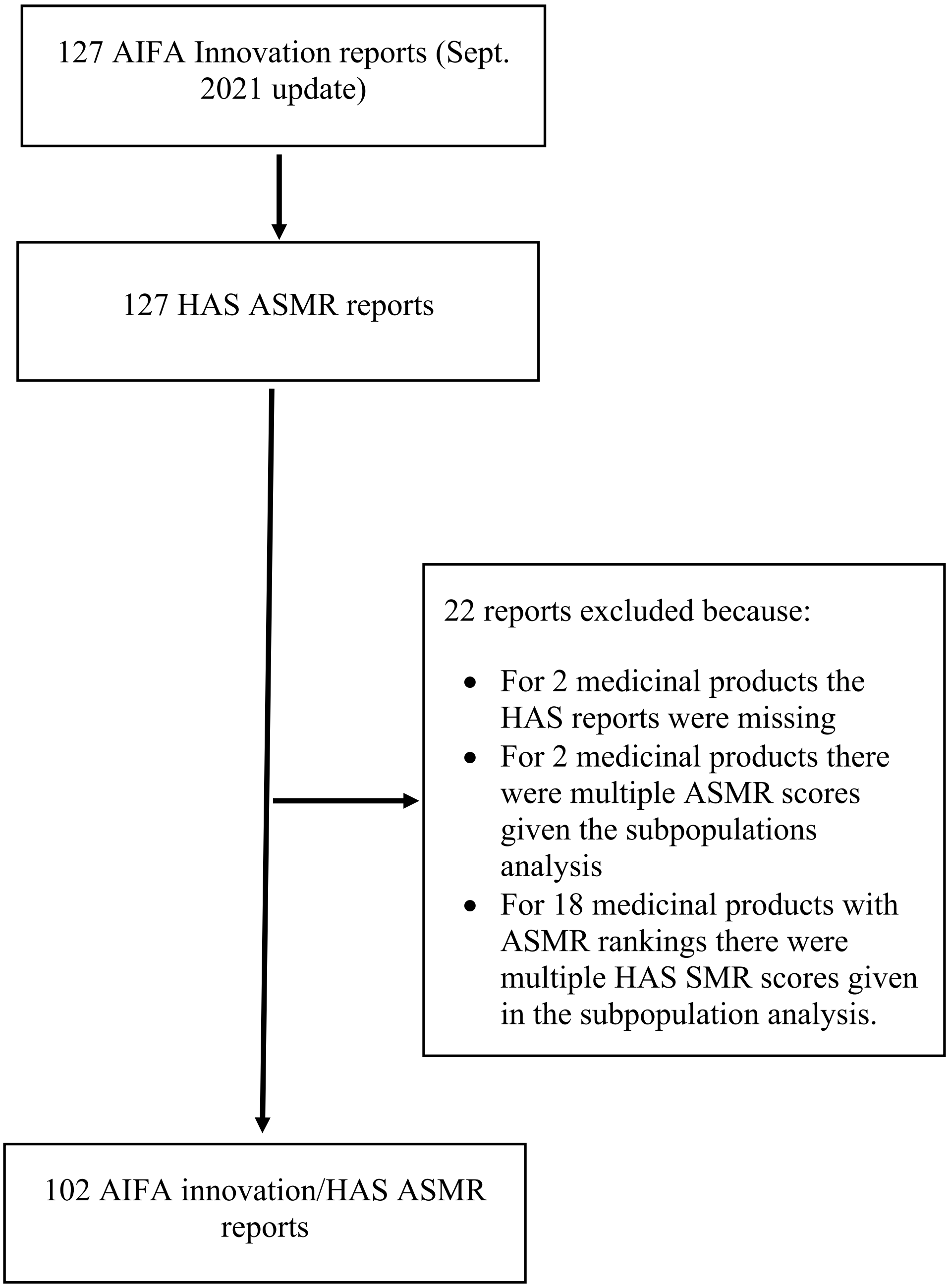


AIFA, Italian Medicines Agency; ASMR, *Amélioration du Service Médical Rendu*; HAS, Haute Autorité de Santé

**Supplementary Figure 2. Comparison of AIFA valuation year vs. HAS**

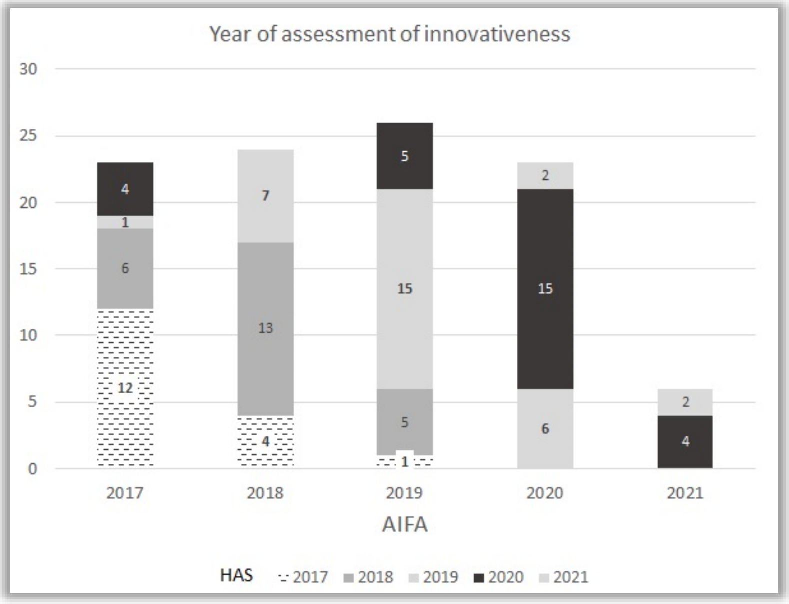


The histogram represents the innovativeness judgments assigned by HAS for the AIFA evaluation years. The AIFA evaluation year is represented on the x-axis, while the number of HAS evaluations is represented on the y-axis.

AIFA, Italian Medicines Agency; ASMR, *Amélioration du Service Médical Rendu*; HAS, Haute Autorité de Santé

**Supplementary Table 1. Interpretation criteria for kappa statistics**

| Value of k | Strength of agreement |
|---|---|
| < 0.2 | Poor |
| 0.21–0.40 | Fair |
| 0.41–0.60 | Moderate |
| 0.61–0.80 | Good |
| 0.81–1.00 | Very good |

**Supplementary Table 2. Distribution of AIFA Innovation status vs HAS ASMR**

| AIFA<br>n° | ASMR I<br>n° | ASMR II<br>n° | ASMR III<br>n° | ASMR IV<br>n° | ASMR V + NA*<br>n° |
|---|---|---|---|---|---|
| Full Innovation status<br>38 | 0 | 4 | 16 | 16 | 2 |
| Conditional Innovation status<br>31 | 0 | 0 | 1 | 22 | 8 |
| No Innovation status<br>33 | 0 | 0 | 1 | 9 | 23 |

AIFA, Italian Medicines Agency; ASMR, *Amélioration du Service Médical Rendu*; HAS, Haute Autorité de Santé

(*) There are 9 cases ASMR NA of which: 1 AIFA fully innovative, 2 AIFA conditional and 6 AIFA non-innovative
